# Identification of a new tospovirus causing necrotic ringspot on tomato in China

**DOI:** 10.1186/s12985-014-0213-0

**Published:** 2014-12-03

**Authors:** Yueyan Yin, Kuanyu Zheng, Jiahong Dong, Qi Fang, Shiping Wu, Lishuang Wang, Zhongkai Zhang

**Affiliations:** Yunnan Key Laboratory of Agricultural Biotechnology, Key Lab of Southwestern Crop Gene Resources and Germplasm Innovation, Biotechnology and Germplasm Resources Institute, Ministry of Agriculture, Yunnan Academy of Agricultural Sciences, Kunming, 650223 China; Institute of Alpine Economic Plants, Yunnan Academy of Agricultural Sciences, Lijiang, 674100 China; Institute of Plant Protection, Guizhou Academy of Agricultural Sciences, Guiyang, 550006 China

**Keywords:** Tospovirus, Tomato necrotic spot virus, S RNA

## Abstract

**Background:**

Emerging tospoviruses cause significant yield losses and quality reduction in vegetables, ornamentals, and legumes throughout the world. So far, eight tospoviruses were reported in China. Tomato fruits displaying necrotic and concentric ringspot symptoms were found in Guizhou province of southwest China.

**Finding:**

ELISA experiments showed that crude saps of the diseased tomato fruit samples reacted with antiserum against Tomato zonate spot virus (TZSV). Electron microscopy detected presence of quasi-spherical, enveloped particles of 80–100 nm in such saps. The putative virus isolate was designated 2009-GZT. Mechanical back-inoculation showed that 2009-GZT could infect systemically some solanaceous crop and non-crop plants including *Capiscum annuum*, *Datura stramonium*, *Nicotiana benthamiana*, *N. rustica*, *N. tabacum* and *Solanum lycopersicum*. The 3012 nt full-length sequence of 2009-GZT S RNA shared 68.2% nt identity with that of Calla lily chlorotic spot virus (CCSV), the highest among all compared viruses. This RNA was predicted to encode a non-structural protein (NSs) (459 aa, 51.7 kDa) and a nucleocapsid protein (N) (278 aa, 30.3 kDa). The N protein shared 85.8% amino acid identity with that of CCSV. The NSs protein shared 82.7% amino acid identity with that of Tomato zonate spot virus(TZSV).

**Conclusion:**

Our results indicate that the isolate 2009-GZT is a new species of *Tospovirus*, which is named Tomato necrotic spot virus (TNSV). This finding suggests that a detailed survey in China is warranted to further understand the occurrence and distribution of tospoviruses.

## Background

Thrips-transmitted tospoviruses cause significant economic losses in tomato, chilli and many other important crops worldwide [1]. At least 13 thrips species have been identified as vectors [2]. Tospoviruses are quasi-spherical, enveloped particles of 80–120 nm in diameter. A tospoviral genome consists of three negative or ambisense ssRNAs designated S, M and L. The S RNA encodes a nucleocapsid protein (N) in viral-complementary sense orientation and the non-structural protein (NSs) in viral sense orientation [3]. NSs acts as a gene-silencing suppressor [4]. The M RNA encodes a non-structural protein (NSm), in the sense orientation, that is involved in cell-to-cell movement and a glycoprotein precursor in complementary sense orientation [5]. The L RNA encodes an RNA-dependent RNA polymerase protein (RdRp) in complementary sense orientation [6].

Among the more than 20 Tospoviruses reported so far [6-10], 8 were found in China. These 8 species include Calla lily chlorotic spot virus (CCSV) [11], Capiscum chlorosis virus (CaCV) [12] , *Groundnut yellow spot virus* (GYSV) [13], Hippeastrum chlorotic ringspot virus (HCRV) [14], *Impatiens necrotic spot virus* (INSV) [15], Tomato zonate spot virus (TZSV) [16], *Tomato spotted wilt virus* (TSWV) [17] and *Watermelon silver mottle virus* (WSMoV) [18]. In 2009, a new tomato disease causing black necrotic and concentric ringspot symptoms on tomato fruits was found in Wengan County, Guizhou province of China. In this study, we identified a new *Tospovirus* species, provisionally named Tomato necrotic spot virus (TNSV), as the causal agent of this tomato disease.

## Material and methods

### Virus source and maintenance

A virus (isolate 2009-GZT) was isolated from naturally infected tomato fruits exhibiting necrotic and concentric ring spots in Wengan County, Guizhou province, China in 2009. The virus was maintained in systemic hosts *Nicotiana benthamiana* and *N. rustica* and in local lesion host *Chenopodium quinoa* by mechanical inoculation as described previously [16].

### ELISA

TZSV-specific antiserum (produced in our laboratory) and ELISA kits for detection of GRSV/TCSV, INSV, IYSV, and TSWV (Agdia, Elkhart, Indiana, USA) were used in ELISA to test saps collected from symptomatic tomato fruits and plants inoculated with isolate 2009-GZT.

### Electron microscopy

Crude plant saps, extracted from natural symptomatic tomato fruits and from plant leaves mechanically inoculated with isolate 2009-GZT, were stained with 2% Ammonium Molybdate for electron microscopy as previously described [16].

### RNA extraction, RT-PCR and sequencing

Total RNA was extracted from symptomatic tomato fruits using TRIzol Reagent (Invitrogen, Carlsbad, CA, USA) according to the manufacturer’s instructions. The universal primer pairs (TospS-3 W:GC(a/t)GTTCCAGGGTT(a/g)CT(t/c/g)TC, and Tosp-3: AGAGCAATCGAGGCGCTAATAA) designed based on the 3ʹ-end sequences of S RNAs of the members of *Watermelon silver mottle virus* (WSMoV) were used to obtain the 3ʹ-end sequence of about 700 nt of 2009-GZT. The cDNA was synthesized using AMV reverse transcriptase (Promega, Madison, Wisconsin, USA). The primer pairs J13 and UHP were used to amplify the NSs and N genes following the protocols described previously [14,19]. The RT-PCR products were purified with UNIQ-10 (Sangon, Shanghai, China) and then cloned into pGEM-T Easy (Promega, Madison, WI, USA). The recombinant clones were sequenced. Based on these sequences, the specific primers (GZ-S1: GTTCAGGACCACCACAAAGGGAT, and GZ-S6R: GCAATCTCTCTGAACAAGTA) were designed to amplify the remaining sequence of S RNA.

### Sequence analysis

The complete sequence of 2009-GZT S RNA was assembled and analyzed with the aid of DNAMAN version 5.0 (Lynnon Biosoft, QC, Canada). Phylogenetic trees were constructed using the neighbor-joining method with 1000 bootstrap replications in MEGA 6.0 [20]. The sequences used for comparison were obtained from the GenBank database: NC_018071(Bean necrotic mosaic virus, BeNMV), NC_008301 (Capsicum chlorosis virus, CaCV), AY867502 (Calla lily chlorotic spot virus, CCSV), NC_003619 (*Groundnut bud necrosis virus*, GBNV), AF080526 (Groundnut chlorotic fan-spot virus, GCFSV), L12048 (*Groundnut ringspot virus*, GRSV), AF013994 (*Groundnut yellow spot virus*, GYSV), KC290943 (Hippeastrum chlorotic ringspot virus, HCRV), NC_003624 (*Impatiens necrotic spot virus*, INSV), AF001387 (*Iris yellow spot virus*, IYSV), EU275149 (Melon severe mosaic virus, MSMV), AB038343 (Melon yellow spot virus, MYSV), KF383956(Pepper chlorotic spot virus, PCSV), EF445397 (Polygonum ringspot virus, PolRSV), HQ728387 (Soybean vein necrosis associated virus, SVNaV), NC_002051(*Tomato spotted wilt virus*, TSWV), AY686718 (Tomato yellow ring virus, TYRV), NC_010489 (Tomato zonate spot virus, TZSV), EU249351 (Watermelon bud necrosis virus, WBNV), NC_003843 (*Watermelon silver mottle virus*, WSMoV) for S RNAs; GQ478668 (Alstroemeria necrotic streak virus, AlNSV), AF067068 (Chrysanthemum stem necrosis virus, CSNV), S54325 (Tomato chlorotic spot virus, TCSV), FJ946835 (Tomato necrotic ringspot virus, TNRV), and AF067069 (*Zucchini lethal chlorosis virus*, ZLCV) for N genes.

## Results

### Virus source, ELISA, virus isolation and host range

**Figure 1:**
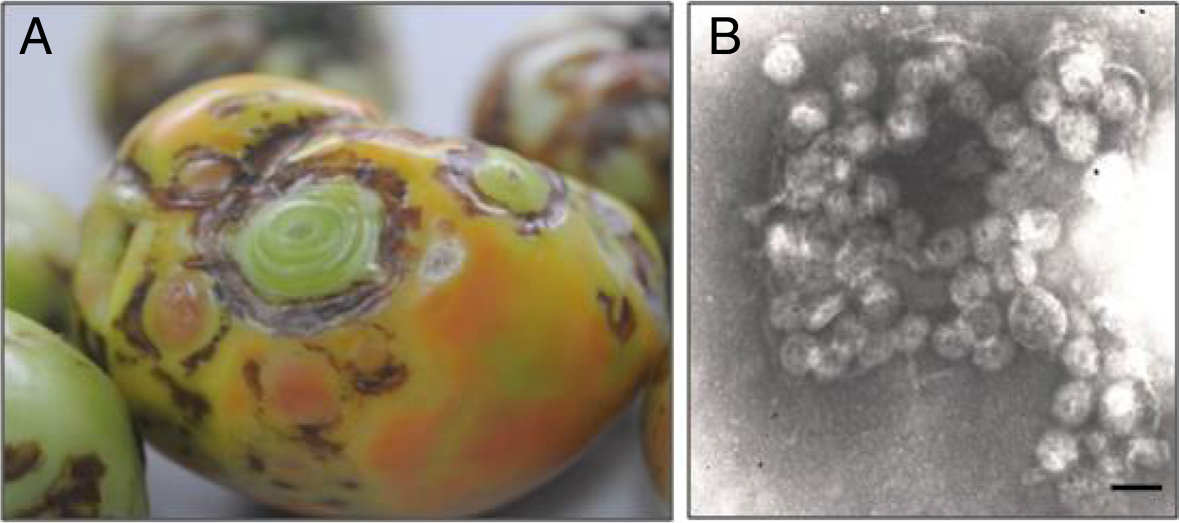
**Symptoms and virus particles of Tomato necrotic spot virus. A**. Necrotic ringspot on tomato fruits. **B**. Electron micrograph of Tomato necrotic spot virus particles (Bar = 100 nm).

Tomato fruit samples showing necrotic ringspot and concentric ringspot symptoms were collected from Wengan County, Guizhou province, China in 2009 (Figure 1A). These samples were tested by ELISA with antisera against GRSV/TCSV, INSV, IYSV, TSWV, and TZSV, respectively. The symptomatic samples reacted with the antiserum against TZSV N protein. The virus isolate, 2009-GZT, was obtained via three consecutive single lesion transfer in *C. quinoa* and in systemically infected *N. rustica* after mechanical inoculation. Further infection studies were used to determine the experimental host range of 2009-GZT. Systemic infection was observed in *C. annuum*, *D. stramonium*, *N. benthamiana*, *N. debneyi*, *N. rustica*, *N. tabacum* and *S. lycopersicum*, with the infected plants showing leaf chlorosis, necrosis, ringspot and/or fruit deformation. Local lesion infection was found in *C. amaranticolor*, *C. quinoa* and *N. glutinosa*. The virus did not infect *Cucumis sativus* (Table 1). ELISA showed that samples from all infected plants reacted positively with the TZSV-antiserum, but did not react with GRSV/TCSV, INSV, IYSV and TSWV antisera.

**Table 1:** **Symptoms induced by Tomato necrotic spot virus on mechanically inoculated plants**

**Family**	**Species**	**Local**	**Systemic**
*Chenopodiaceae*	*Chenopodium amaranticolor*	NL	—
	*C. quinoa*	NL	
*Cucurbitaceae*	*Cucumis sativus*	—	—
*Solanaceae*	*Datura stramonium*	NL	NSCRS
	*Solanum Lycopersicum*	NL	BN
	*Nicotiana benthamiana*	NL	BN
	*N. rustica*	NLCRS	CRS
	*N. glutinosa*	NL	—
	*N. debneyi*	M, NL	M, NL
	*N. tabacum* var Honghuadajinyuan	NL	AN
	*N. tabacum* var *K326*	NL	AL
	*Capiscum annuum* var Hongxianfeng (sweet pepper)	NL	CRS
	*C. annuum var* (chilli pepper)	NL	M, CRS
*Asteraceae*	*Lactuca sativa* L. var ramosa Hort	N	—
	*Lactuca sativa* L. var longifoliaf. Lam	N	—

N: necrosis; NL: necrotic lesion; CRS: Chlorotic ring spot; BN: Bud necrosis; M: mosaic; —: nonsymptom.

### Electron microscopy

Roughly spherical and enveloped virions of 80–100 nm in diameter, typical of tospoviruses, were observed in the saps collected from 2009-GZT-infected *N. tabacum* var Honghuadajinyuan by electron microscopy (Figure 1B).

### Molecular characterization and sequence analysis of 2009-GZT S RNA

The complete nucleotide sequence of the 2009-GZT S RNA contained 3012 nt (accession no. KM355773). It had NSs and N open reading frames (ORFs) in an ambisense orientation, separated by a 670 nt intergenic region (IGR) with an AU content of 72.9%. The 1380 nt NSs ORF, from nt 67 to 1446 of the viral RNA (vRNA) strand, potentially encoded a protein of 459 amino acids. The 828 nt N ORF, from nt 2112 to 2945 of the viral complementary RNA strand, potentially encoded a protein of 276 amino acids. The predicted molecular masses of NSs and N proteins were 51.6 kDa and 30.2 kDa, respectively. The 5′- and 3′-untranslated regions were 66 and 68 nt long, respectively.

The 2009-GZT S RNA sequence shared the highest (68.2%) and lowest (28.9%) nucleotide identities with those of CCSV and GYSV, respectively. The N gene and protein shared higher nucleotide (60.2-76.3%) and amino acid (69.9-85.8%), respectively, sequence identities with those of the WSMoV serogroup (WSMoV, CaCV, GBNV, WBNV, TZSV and CCSV) than with those of the IYSV serogroup (IYSV, TYSV, HCRV and PolRSV) (53.5-55.7% nucleotide and 46.1-48.9% amino acid identities). It shared relatively low nucleotide (36.1-45.3%) and amino acid (16.4-36.1%) identities with those of GYSV, GCFSV, INSV, and those of the TSWV serogroup members (Table 2).

**Table 2:** **Comparisons of the sequence identities of S RNA and deduced proteins of Tomato necrotic spot virus with those of other Tospoviruses**

**Virus**	**S RNA**		**5′UTR nt**	**NSs protein aa %**	**IGR nt**	**N protein aa %**	**3′UTR nt**
	**nt**	**nt %**					
TNSV	3012	100	66	100	670	100	68
SVNaV	2603	36.3	58	16.8	320	36.1	71
BeNMV	2584	37.8	60	23.2	316	30.9	76
CaCV	3477	55.0	66	61.6	1202	70.3	67
CCSV	3172	68.2	66	80.8	831	85.8	65
GRSV	3049	38.6	87	18.4	636	24.2	152
PCSV	2786	58.1	65	57	449	53.4	67
GYSV	2970	28.9	57	14.9	653	16.4	77
INSV	2992	32.9	62	16.5	648	24.2	50
IYSV	3105	54.1	70	52.3	817	48.9	71
MYSV	3232	59.8	68	48.5	853	61.9	68
GBNV	3057	63.1	66	64.2	779	70.3	68
GCFSV	2833	30.7	67	14.4	461	17.4	80
TSWV	2916	38.8	88	17.1	509	23.3	54
TYRV	3061	54.6	71	52.0	768	47.0	72
TZSV	3297	67.0	64	82.7	934	81.7	65
WBNV	3405	57.0	66	63.1	1124	71.2	18
WSMoV	3534	57.8	67	61.8	1261	69.9	66
PolRSV	2484	54.4	72	51.2	183	46.1	71
MSMV	3283	38.4	80	17.9	887	27.4	159
HCRV	2744	58.5	73	51.2	437	46.6	71
ANSV	—	—	—	—	—	25.1	—
CSNV	—	—	—	—	—	24.7	—
ZLCV	—	—	—	—	—	23.3	—
TCSV	—	—	—	—	—	24.7	—
TNRV	—	—	—	—	—	61.9	—

**Figure 2:**
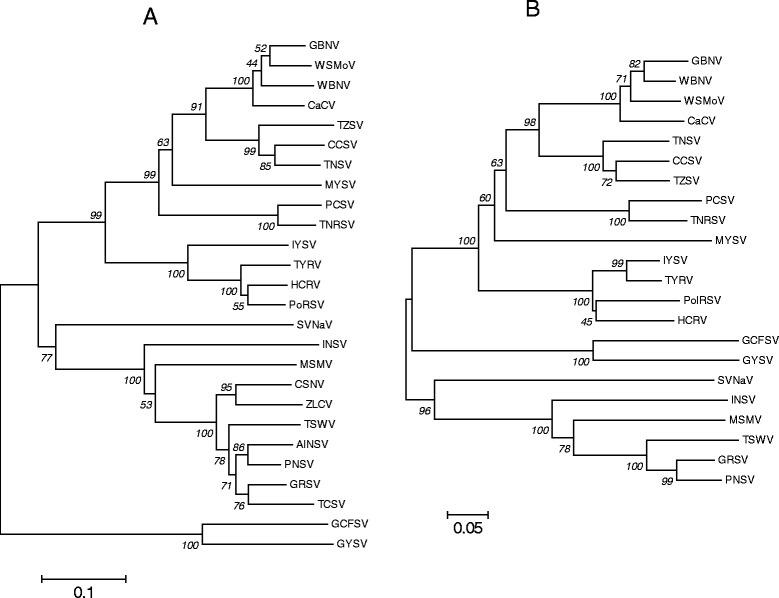
**Phylogenetic analyses of Tospoviruses based on amino acid sequences of the nucleocapsid (N) protein (A) and the non-structural (NSs) protein (B).** The analyses were conducted using MEGA6.0 software. Bootstrap values on the branches represent the percentage of 1,000 bootstrap replicates.

The NSs gene shared relatively higher identities (58–76.3% nucleotide and 61.6-80.8% amino acid) with those of the WSMoV serogroup than those of the other tospoviruses (Table 2). The S RNA IGR sequence shared identities (40.3%-52.3%) with those of the other tospoviruses. Phylogenetic analyses showed that the 2009-GZT N and NSs proteins were closely related to those of CCSV and TZSV, thereby clustered into the WSMoV serogroup (Figure 2).

## Discussion

Although 2009-GZT was originally isolated from tomato, it could infect other solanaceous species. The viral symptoms in those host plants were similar to those described for other tospoviruses such as TSWV, TZSV and HCRV. The virion morphology, ELISA results, and S RNA sequence characteristics indicate that isolate 2009-GZT is a tospovirus. The S RNA sequence divergence between 2009-GZT and the other tospoviruses is at the inter-species level (Table 2 and Figure 2) [6]. Thus, we propose 2009-GZT as a new species of *Tospovirus* with a provisional name Tomato necrotic spot virus (TNSV). During the field survey, *Thrips tabaci* and *T. palmi* were found in tomato fields and the surrounding weeds. These two thrips species may represent potential vectors of TNSV.

Infection by tospoviruses has emerged as an important constraint to the productivity and quality of crops in many regions of the world [2,21,22]. Since 1980’s, emergence and geographic expansion of new variants of known tospoviruses and completely new tospoviruses have been occurring in many regions of China, including Beijing, Guangdong, Guangxi, Hebei, Shandong, Sichuan and Yunnan [12,16,18,23,24]. This is first report of occurrence of tospovirus disease in Guizhou province. While emergence of tospovirus diseases is a multifactorial process (including virus, vector, host, environment, and human behavior) and remains poorly understood, presence of tospoviruses-transmitting thrips (*Frankliniella occidentalis*, *F. intonsa*, *T. palmi*, and *T. tabaci*) [25] and large scale plantation of tospovirus-susceptible crops (tomato, chilli and tobacco) suggest potential occurrence of tospoviral diseases in other regions of China. So, a more detailed tospovirus survey in China is needed to understand the occurrence and distribution of tospoviruses and to develop measures preventing the epidemic of tospovirus diseases.

## Abbreviations

nt: Nucleotides
aa: Amino acids
N: Nucleocapsid protein
RdRp: RNA-dependent RNA polymerase protein
ELISA: Enzyme Linked Immunosorbent Assay
